# Improvement of Pain and Function by Using Botulinum Toxin Type A Injection in Patients with an Osteoarthritic Knee with Patellar Malalignment: An Electromyographic Study

**DOI:** 10.3390/life13010095

**Published:** 2022-12-29

**Authors:** Alice Chu Wen Tang, Chih-Kuang Chen, Szu Yuan Wu, Simon F. T. Tang

**Affiliations:** 1 Department of Physical Medicine and Rehabilitation, Fu Jen Catholic University Hospital, New Taipei City 234, Taiwan; 2 Department of Physical Medicine and Rehabilitation, Linkou Chang Gung Memorial Hospital and Chang Gung University, Taoyuan 333423, Taiwan; 3 Division of Radiation Oncology and Big Data Center, Lo-Hsu Medical Foundation, Lotung Poh-Ai Hospital, Yilan 265501, Taiwan; 4 Graduate Institute of Business Administration, College of Management, Fu Jen Catholic University, Taipei 24205, Taiwan; 5 Centers for Regional Anesthesia and Pain Medicine, Taipei Municipal Wan Fang Hospital, Taipei Medical University, Taipei 11031, Taiwan; 6 Department of Physical Medicine and Rehabilitation, Lotung Poh-Ai Hospital, Yilan 265501, Taiwan

**Keywords:** osteoarthritic knee, patellar malalignment, patellofemoral pain syndrome, electromyography, vastus medialis oblique, vastus lateralis botulinum toxin type A injection

## Abstract

Objective: To determine the pain and electromyographic (EMG) amplitude ratio of the vastus medialis oblique (VMO) to the vastus lateralis (VL) after botulinum toxin type A (BTA) was injected in the bilateral osteoarthritic knee of patients with patellar malalignment for analysis. Material and methods: A total of fifteen patients were recruited; the more symptomatic knee of each patient received a BTA injection (BTA side). The other set of patients were left untreated. In all, fifteen healthy participants comprised the control group. The Western Ontario and McMaster Universities Osteoarthritis Index (WOMAC) and numeric rating scale (NRS) for pain were assessed. The EMG amplitude of VL and VMO activity was recorded using an isokinetic dynamometer and synchronized using the BIOPAC MP100. The data were collected before and at 4, 8, and 12 weeks post–BTA injection. Results: The EMG ratios of the patient group were lower than those of the control group at all testing velocities (*p* < 0.05). The VMO/VL ratio improved significantly on the BTA side only. The VMO/VL ratios on the BTA side were higher than those on the untreated side (*p* < 0.05). Knee pain decreased significantly after the BTA injection. The EMG ratios were negatively correlated with the NRS and WOMAC scores. Conclusion: BTA injection effectively reduces knee pain and restores the EMG ratio between the VMO and VL.

## 1. Introduction

Among older adults, the most prevalent type of arthritis and a leading cause of disability is knee osteoarthritis (OA) [1]. Knee OA is frequently evaluated through radiographic assessments, in which joint space narrowing and cartilaginous degeneration are localized to enable classification of the OA as tibiofemoral (TF) or patellofemoral (PF) [2]. Subsequently, patients with knee OA that have combined TF/PF OA or OA were isolated to the medial TF compartment. In patients with combined OA, merchant view radiographic assessment often reveals the presence of patellar lateral subluxation (PLS) [2,3,4,5]. PLS is the translational displacement of the patella relative to the femoral trochlea that often involves a tilt. However, patients with knee OA with PLS and patellofemoral pain syndrome (PFPS) have similar clinical features, namely, anterior knee pain (AKP) with a notable impairment of the ability to climb and descend stairs and sudden pain when rising from a chair. Therefore, hereafter, we use PFPS to represent knee OA with PLS. In cases of PFPS, arthrosis may occur because the tilt and subluxation associated with the disease change the loading of the articular cartilage of the patellofemoral joint [6]. Young women and athletes are most prevalently affected by AKP [1,2,3]. Some authors have reported that AKP may result in articular degeneration in late life [7,8].

The conservative treatment was the main initial management for PFPS, while surgical treatment was reserved for patients who not only failed the conservative treatment for >6 months but also with documented malalignment, a tight lateral retinaculum, or an articular cartilage lesion [9]. Although some researchers have reported positive results for conservative treatments, including those involving knee braces [10], taping [11], VMO muscle strengthening [12], electrical muscle stimulation [13], and foot orthotics [14,15], few high-quality randomized controlled trials have been conducted to verify the effectiveness of such treatments [16]. Furthermore, recent systemic reviews and meta-analyses showed that limited evidence supported the efficacy of conservative treatments [17].

PLS mainly results from abnormal bony structures and lower extremity muscle imbalance. Abnormal bony structures are more likely associated with the femur bone having a flattened trochlear groove, pronation, or forward tilting [5,6,18,19,20]. Whereas the aforementioned muscle imbalance is most often the result of vastus lateralis (VL) and vastus medialis oblique (VMO) muscle tone imbalance. The vastus medialis oblique (VMO) muscle has been addressed as “the only medial dynamic stabilizer” by McConnell [21] and is composed of type I fiber (slow-switch fiber) up to 52.1% [22]. Apart from traditional conservative treatment and surgical management, Botulinum toxin type A(BTA) injections attracted attention in treating PFPS by their ability to weaken selected muscles [23]. Botulinum toxin type A(BTA) prevents acetylcholine secretion in the neuromuscular junction, and an animal model showed that 3–4 months after the injection, the neuromuscular transmission recovered with the formation of motor axon sprouts and synaptic contact [24]. Recent studies showed that BTA also caused pain relief by reducing peripheral sensitization [25,26], decreasing the inflammatory signal in induced-arthritis animal models [27,28], and exerting a central effect by changing sensory afferents [29,30]. Therefore, BTA injection is promising in treating PFPS because of its direct effect on regulating muscle contraction, additional analgesic effect, and reversible trait. However, previous literature has discussed the use of BTA injection in PFPS [23,24,25,26,27,28,29,30,31,32,33]. Several studies suggest that a single BTA injection significantly reduced pain and improved function in PFPS patients [23] Other studies identified several electromyographic features in PFPS patients, including delayed activation of VMO [34] and reduced peak extension movement [35,36]. However, the effect of BTA injection on altering VL and VMO activation remained unclear.

The present study used surface EMG to conduct a comparison of EMG ratios between the VMO and VL before and after BTA injection to thoroughly investigate the effects of BTA in treating PLS-related AKP.

## 2. Materials and Methods

### 2.1. Participants

This research was approved by the institutional review board of a tertiary medical center (IRB number: 101-5028A3). All the patients signed informed consent forms before participation. Additionally, fifteen patients with symptomatic osteoarthritis of the knee were recruited from an outpatient clinic between 2013 and 2016. Further, anteroposterior radiographic assessment was employed to assign each case of knee OA an Ahlbäck classification grade of 1 or 2. Merchant’s view was used to identify patellar malalignment. In the experimental group, the knee with the worst pain was selected for BTA injection (BTA side). The other knee remained untreated (untreated side).

The exclusion criteria were as follows: (1) an inability for independent walking; (2) no Merchant’s view–identifiable PLS or tilting; (3) neurological comorbidities, including multiple sclerosis, Alzheimer disease, or Parkinson disease; (4) rheumatoid arthritis or other inflammatory diseases of the joints; (5) a history of previous lower extremity joint (e.g., hip, knee, or ankle) injury; (6) severe medial tibia degeneration, as indicated by a Kellgren–Lawrence or Ahlbäck’s grade higher than II; (7) previous surgery related to the lower extremities, including amputation, replacement, or ligament repair; (8) a BTA allergy, plan to attempt a pregnancy within 2 years, or current pregnancy; and (9) an inability to comprehend or complete the experiment.

The fifteen healthy participants without any knee pain symptoms and normal alignment of the patella, according to the merchant’s view, were recruited as a healthy control group. The experimental and control groups exhibited no significant differences in age, height, or body mass index (*p* > 0.05; Table 1).

### 2.2. Injection of BTA

The study participants were placed in the supine position. The knee with more severe pain was selected for BTA injection, and the other knee did not receive treatment. Normal saline was used to dilute a BOTOX 100 U/vial (Allergan, Irvine, CA, USA) to a concentration of 10 U/0.1 mL. The VL was located using a 27-gauge open-lumen needle that was Teflon coated. The repetitive square wave pulses were applied once per second for a duration of 0.25 ms to the targeted muscle at a location 3 to 5 cm above the patella and lateral to the midline at an oblique angle [37]. A notable lateral shift of the patella occurred after the pulses were applied when the open lumen needle was placed on the VL.

### 2.3. Clinical Assessment

The consent of each participant was sorted and a profile survey and clinical questionnaire were completed. The profile survey was used to obtain each participant’s name, sex, age, height, and weight. The participants’ knee stiffness, pain, and functional status were determined using the Western Ontario and McMaster Universities Osteoarthritis Index (WOMAC). On the WOMAC, of the 96 possible points, 68 points represented functional status, 20 points represented pain severity, and 8 points represented stiffness level. Numerous studies have reported on the responsiveness and sensitivity of the WOMAC, and the WOMAC’s function and pain subscales have been reported to demonstrate a higher responsiveness to change than the function and pain subscales of the SF-36 [38,39]. An 11-point numeric rating scale (NRS) was used to assess knee pain.

### 2.4. Isokinetic Test

A Cybex Norm (Cybex International, Medway, MA, USA) dynamometer was used to complete an isokinetic concentric assessment of the extensor muscles of the knee. To test the flexors and extensors, the participants were asked to assume the seated position on the Cybex dynamometer. The backs of the participants were supported, and their hips were flexed to an angle of 85° [40]. Additionally, the trunk and thighs were stabilized using straps. The test velocity was set at 60, 120, and 180 rad/s, and the knee joint angular movement was within the range of 90° to 0°. Further, each participant was asked to walk at submaximal contraction levels for 10 min and then rest in the sitting position for 5 min. During the tests, the participants completed 5 isokinetic concentric contractions from 90° (flexion) to 0° (extension) at 60, 120, and 180 rad/s. Visual feedback was used to encourage the participants to perform maximal contractions.

### 2.5. EMG Assessment

The EMG signals were obtained using a BIOPAC MP100 system with six channels (BIOPAC Systems, Goleta, CA, USA). A total of four channels were used to record direction (flexion/extension), angular velocity, position (degree of knee extension), and torque signals, while two channels were applied to record the muscle activity amplitude. The skin of the participants was prepared using isopropyl alcohol, and bipolar gold-plated surface electrodes were placed over the center of the VMO and distal VL muscle bellies at a 3-cm interelectrode distance, as specified in Basmajian and Blumenstein [41]. The ground electrode was placed over the tibial tubercle. The participants’ peak muscle torque and EMG activity were recorded when the knee extension tests were completed. The data were collected at 1000 Hz and stored using AcqKnowledge software (version 3.7.3) on a personal computer. The data were subsequently analyzed using custom LabVIEW software (Version 7.1, National Instruments Corporation, Austin, TX, USA). The EMG linear envelope method was used to process the raw EMG data. The signals were bandpass filtered (20–500 Hz), full-wave rectified, and smoothed at 6 Hz by using a low-pass second-order Butterworth digital filter. The peak amplitudes of the filtered VMO and VL EMG signals are presented as VMO/VL ratios. The VMO/VL ratios were averaged for each velocity trial over three extension cycles, with the first and fifth cycles excluded. A ratio of less than 1 was considered to indicate that the EMG signal activity for the VMO was lower than that for the VL.

### 2.6. Statistical Analysis

The statistical analysis was conducted using SPSS 21.0 for Windows (SPSS, Chicago, IL, USA). The demographic data and the VMO/VL ratios were compared using a Student’s t test, while the sex ratios of the patients and normal participants were analyzed using a chi-squared (χ^2^) analysis. The BTA and untreated sides at four time points (before, after 4 weeks, after 8 weeks, and after 12 weeks) and their interactions with respect to the VMO/VL ratios were compared by using a two-way repeated measures analysis of variance. After the main effects were detected to have significant differences, Bonferroni-corrected paired *t* tests were applied as post hoc tests. Furthermore, a Friedman test was performed in order to analyze the effect of time points on WOMAC scores. If the Friedman test result was significant, Wilcoxon’s signed-rank test was performed between both time points and between the BTA side and the untreated side. The Spearman correlation test was conducted to determine the association between two variables measured on an ordinal scale. Significance was considered to have occurred when *p* < 0.05.

## 3. Results

A comparison of the demographic data of the PFPS and normal groups is presented in Table 1. No significant differences were observed between the groups.

### 3.1. VMO/VL Ratio before and after BTA Injection

**Figure 1:**
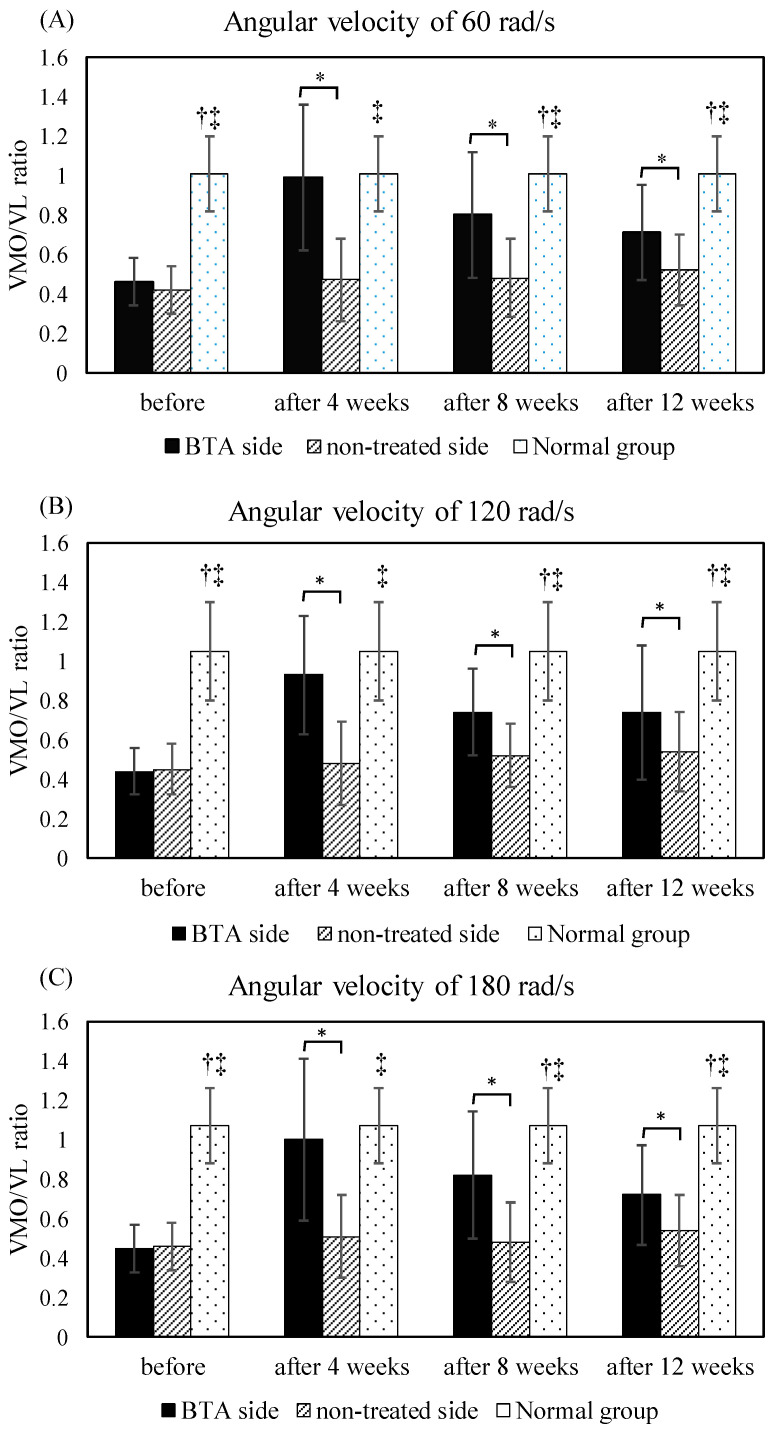
Comparisons of VMO/VL ratio between BTA and untreated sides and between patients and healthy controls at angular velocities of (**A**) 60, (**B**) 120, and (**C**) 180 rad/s. *: *p* < 0.05, knees of BTA side compared with the untreated side; †: *p* < 0.05, knees of healthy controls compared with the BTA side; ‡: *p* < 0.05, knees of healthy controls compared with untreated side; BTA: botulinum toxin type A.

Figure 1 illustrates the differences in the VMO/VL ratios among the BTA side, the untreated side, and the knees of the normal control group. The VMO/VL ratio was significantly larger in the knees of the normal control group than it was in the BTA side and the untreated side at angular velocities of 60, 120, and 180 rad/s before the BTA treatment and at 8 and 12 weeks after the BTA treatment (*p* < 0.05). Notably, no significant difference was identified between the knees of the normal control group and the BTA side at angular velocities of 60, 120, and 180 rad/s at 4 weeks after the BTA treatment. The VMO/VL ratio was significantly larger for the BTA side than for the untreated side at angular velocities of 60, 120, and 180 rad/s at 4, 8, and 12 weeks after BTA treatment (*p* < 0.05).

### 3.2. WOMAC Questionnaire and NRS

**Figure 2:**
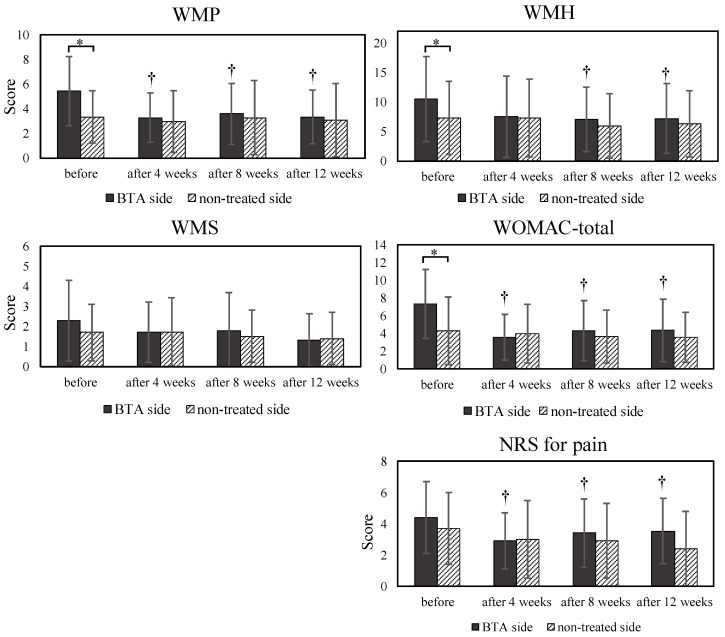
Comparisons of pain, stiffness, and functional limitation subscales of WOMAC, WOMAC-total, and numerical rating scale (NRS) scores. WMP: pain subscale of the WOMAC; WMS: stiffness subscale of the WOMAC; WMH: functional limitation subscale of the WOMAC; *: *p* < 0.05, between the BTA side and untreated side; †: *p* < 0.05, comparison with before; BTA: botulinum toxin type A.

Figure 2 illustrates the associations between the VMO/VL ratio and multiple clinical indicators, namely the pain subscale of the WOMAC, WOMAC-total, and NRS scores. For the BTA side, the total WOMAC and NRS scores improved significantly over the course of 12 weeks (*p* < 0.05).

### 3.3. Associations of EMG Ratios with WOMAC and NRS

**Figure 3:**
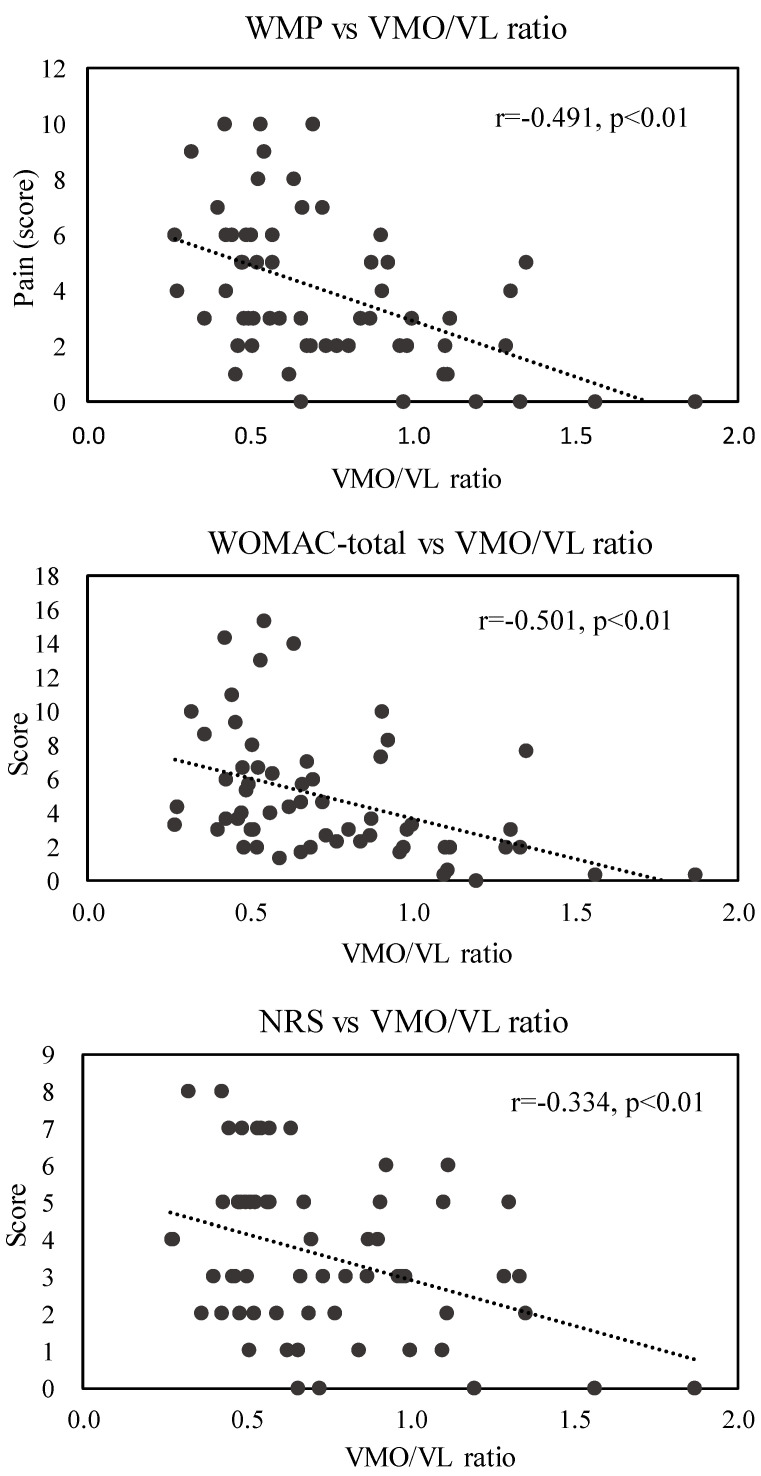
Associations of WMO/VL ratio at 60 rad/s with WMP, WOMAC-total, and NRS scores; WOMAC: Western Ontario and McMaster Universities Osteoarthritis Index; WMP: pain subscale of WOMAC; NRS: numerical rating scale.

The Spearman correlation coefficients for the associations between the total WOMAC scores and the VMO/VL ratios were obtained. The results indicated a negative correlation between the VMO/VL ratios and WOMAC scores (r = 0.501, *p* < 0.01) and a negative correlation between the VMO/VL ratios and NRS scores (r = 0.334, *p* < 0.01; Figure 3).

## 4. Discussion

In the healthy participants without AKP, the EMG ratio between the VMO and VL was approximately 1.01–1.11 at angular velocities of 60, 120, and 180 rad/s. The result was similar to Wu’s finding [41]. In the PFPS group, the EMG ratio between the VMO and VL was approximately 0.46–0.48. The present study agreed with previous research that pain and function were significantly improved after BTA injection [23]. Furthermore, the results demonstrated that the EMG signal imbalance between the VMO and VL was restored through BTA injection, while VMO/VL ratios were negatively correlated with NRS pain scores, suggesting that the malalignment induced friction was decreased by the restoration of VMO/VL ratios. Few studies have observed the change of the EMG signal in PFPS after intervention. One study investigating the effect of patellar taping on the EMG activity of VMO and VL revealed that tapping reversed the activation sequence in PFPS; earlier VMO activation was observed, while the amplitude of VMO and VL did not change [42]. These results suggested that conservative treatment might modulate the activation time of VMO muscle, but the underlying muscle imbalance remained unsolved. On the contrary, BTA injections directly ameliorate the VMO/VL ratios, achieving normalization of quadriceps contraction.

In addition to the pain and functional loss caused by PFPS, it is also a risk factor predisposing to the development of patellofemoral OA, which is an under-recognized subgroup of knee OA [43]. Therefore, correction of malalignment before cartilage damage occurs is critical. Our finding suggested that with effective intervention by BTA injection, the preservation of the patellofemoral joint might be achievable.

The infrapatellar fat pad is a highly innervated area and a potential source of PFPS [43]. Interestingly, it is also a great reservoir for mesenchymal stem cells. Recent studies showed that infrapatellar fat pad-derived mesenchymal stem cells are promising for articular cartilage regeneration [44]. In future work, more detailed stratification of PFPS patients should be considered. In young patients with an intact PF joint, the restoration of VMO/VL by BTA injection might suffice as a treatment goal, while for older patients with evident PF OA, additional regenerative techniques should be explored.

BTA injections are generally used to treat problems related to focal muscle overactivity, including axial or focal dystonia, which often occur in adults [45] and children [46] who experience brain injury. Several extensive uses in nociceptive arthritis pain and neuropathic pain are being discovered nowadays. Animal studies in horses showed intraarticular BTA injection decreased lameness [47], and a systemic review recruiting 5 RCT articles with a total of 314 patients elucidated that intraarticular BTA injection effectively reduced pain and improved WOMAC questionnaire score without adverse events [48]. Additionally, among various factors associated with the pathophysiology of knee OA, NF-κB signaling plays an important role in regulating articular homoeostasis and the catabolic cascade [49]. The transcription factor NF-κB mediates the inflammatory response through pro-inflammtory gene induction, innate and adaptive immune function adjustment, and inflammasome regulation [50]. Excessive mechanical loading in articular cartilage induces Gremlin-1, an extracellular antagonist of bone morphogenetic proteins (BMP), and then activates NF-κB signaling [51]. Botulinum toxin was reported to prevent nociceptive neurotransmitter release in peripheral terminal [52], such as glutamate and substance P [53]. One study that combined the use of Botulinum toxin and minocycline to treat neuropathic pain found that apoptosis, inflammation, and oxidative stress were attenuated in a spinal cord injury-induced rat model, and notably, expression of SIRT1 was promoted, which inhibited the NF-κB pathway [54]. Articular chondrocytes conduct cell-to-cell communication through connexin channels, a transmembrane protein constituting gap junctions that permit ions, nutrients, and second messengers to pass through. Increased expression of Connexin 43 is found in the OA knee [55,56]. One study showed that CX 43 gene expression could be modulated by NF-κB binding to the promotor region [57]. In contrast, another study demonstrated that downregulation of Connexin 43 inactivated the NF-κB pathway [58]. These results might suggest that NF-κB and Connexin 43 interact with each other in complex ways. Therefore, in OA patients with PLS, concomitant use of intramuscular and intraarticular injections of BTA might further improve therapeutic effect.

BTA injection techniques have evolved from anatomical guides to electrostimulation guides and then ultrasound guides, but despite the improvement in guidance precision, it is still an invasive procedure. Liposome-encapsulated BTA has recently been developed to deliver BTA non-invasively to treat functional bladder disorders with promising results. Liposomes successfully carry BTA across the uroepithelium and act on nerve endings [59,60]. However, adequate vehicles to transport BTA to act on the neuromuscular junction have not yet been developed, to our knowledge and according to our literature review.

The safety issue and excessive muscle weakness have been major concerns in BTA injection. Singer et al. reported that BTA injections improved participants’ ability to produce extensor isometric force at 30° flexion (*p* < 0.02) when they completed a timed stair climbing task (*p* < 0.002) [31]. In our previous study, the BTA injection to the VL did not impair participants’ ability to produce quadriceps force or lead to any adverse events [61].

In conclusion, our study demonstrated that BTA injection was safe and effective in restoring VMO and VL muscle balance and treating clinical symptoms of PFPS; further work should be targeted at managing existing OA change by regenerative technique or intraarticular BTA injection. However, there were still several limitations to this study, including the relatively small sample size and the lack of radiographic image comparison before and after BTA injection.

## Figures and Tables

**Table 1:** Comparisons of demographic data between both groups.

	Groups	PFPS Group (*n* = 15)	Normal Group (*n* = 15)	*p* Value
Demographic Data				
Male/Female		1/14	2/13	0.543
Age (years)		47.5 ± 9.5	50.3 ± 5.5	0.322
Body height (cm)		159.9 ± 7.8	159.2 ± 6.5	0.783
Body mass (kg)		60.3 ± 12.3	59.7 ± 10.4	0.874
BMI		23.4 ± 3.2	34.4 ± 3.0	0.986

BMI: body mass index

## Data Availability

The data supporting the reported results can be found in the Gait Laboratory of Chang Gung Memorial Hospital at Taoyuan City.
